# SARS-CoV-2 RNA Presence in Outdoor Air of Public Spaces in Valladolid During Winter, 2021

**DOI:** 10.1007/s12560-024-09615-1

**Published:** 2024-11-30

**Authors:** Priscilla Gomes da Silva, José Gonçalves, Elisa Rodriguéz, Pedro A. García-Encina, Maria São José Nascimento, Sofia I. V. Sousa, João R. Mesquita

**Affiliations:** 1 ICBAS – School of Medicine and Biomedical Sciences, Porto University, Porto, Portugal; 2 Epidemiology Research Unit (EPIunit), Institute of Public Health, University of Porto, Porto, Portugal; 3 Laboratório Para a Investigação Integrativa E Translacional Em Saúde Populacional (ITR), Porto, Portugal; 4 LEPABE – Laboratory for Process Engineering, Environment, Biotechnology and Energy, Faculty of Engineering, University of Porto, Porto, Portugal; 5 ALiCE – Associate Laboratory in Chemical Engineering, Faculty of Engineering, University of Porto, Porto, Portugal; 6 MARE - Marine and Environmental Sciences Centre, ARNET - Aquatic Research Network Associate Laboratory, NOVA School of Science and Technology, NOVA University Lisbon, Caparica, Portugal; 7 Institute of Sustainable Processes, Valladolid University, Valladolid, Spain; 8 Department of Chemical Engineering and Environmental Technology, University of Valladolid, Valladolid, Spain; 9 Faculty of Pharmacy, University of Porto, Porto, Portugal

**Keywords:** SARS-CoV-2, Airborne SARS-CoV-2, Airborne transmission, COVID-19, Public health

## Abstract

As SARS-CoV-2 continues to evolve and herd immunity establishes, an increasing number of asymptomatic infections have been reported, increasing the risk of airborne spread of the virus. Most of the studies regarding SARS-CoV-2 RNA presence in air refer to indoor environments, with few studies having reported SARS-CoV-2 RNA in outdoor air. The aim of this study was to assess the presence of SARS-CoV-2 RNA at two different settings, crowded outdoor versus empty outdoor environments in Valladolid, Spain, during winter 2021. Using a Coriolis® air sampler, samples were taken from nine different locations within the city center. RNA extraction and a one-step RT-qPCR were carried out. Six out of the 20 air samples were found to be positive, and they were all obtained from crowded outdoor environments. These results highlight that although in less quantity, SARS-CoV-2 RNA is still present in outdoor air, especially at moments of relaxed mitigation efforts and depending on the number of people present.

## Introduction

Three years have passed since the World Health Organization (WHO) declared the COVID-19 pandemic (WHO, 2023a), and even though there are now highly effective vaccines and high vaccination coverage in most parts of the world (WHO, 2024), new waves of COVID-19 are still emerging worldwide, mainly due to breakthrough infections in vaccinated individuals and reinfections (Doke et al., 2023). These waves are driven mainly by the rise of new variants of concern such as Omicron (B.1.1.529), which are more transmissible and can evade our immune system more easily (Tan et al., 2023). As of May 2023, the XBB.1 descendant lineages (XBB.1.5, XBB.1.16, and XBB.1.9) predominate globally (WHO, 2023b).

The WHO reported three modes of transmission for SARS-CoV-2 infection: through respiratory droplets, aerosols and fomites (WHO, 2021). SARS-CoV-2 is transmitted from person to person either by direct (person-to-person contact and droplet spread) or by indirect contact with surfaces (fomites) where virus-containing droplets expelled by an infected person have been deposited (Morawska & Cao, 2020), whereas airborne transmission can happen via droplets or aerosols generated during coughing, sneezing, talking, singing or breathing (Jones & Brosseau, 2015). This airborne spread of an infectious pathogen is caused by the dissemination of aerosols that remain infectious when suspended in air over long distances and periods of time (WHO, 2020b).

The probability of airborne transmission is contingent on several still-uncertain parameters, including virus-laden aerosol concentrations, viability and longevity, and the minimum infectious dose required for infection (Buonanno et al., 2020; Contini & Costabile, 2020). Three factors significantly affect the viral load of aerosols discharged from a SARS-CoV-2 infected person: when disease has started; the severity of the illness; and physiological characteristics that control the rate at which aerosols and therefore viral particles are emitted (Cox et al., 2023). Nonetheless, viral dispersion strongly influences SARS-CoV-2 transmission (Poydenot et al., 2022), thus, experimental studies of aerosol dispersion would help avoid and control future public health emergencies (Fen et al., 2023).

Still, as SARS-CoV-2 keeps changing and herd immunity builds, more and more cases of asymptomatic infections are reported, making airborne transmission more likely (Lee et al., 2020). It is also important to consider the role that asymptomatic cases play in the spread of COVID-19 in the community (Gao et al., 2021), as it is particularly important in crowded places, where the chance of transmission significantly increases (Zhang et al., 2023).

In one study, about half of patients who were diagnosed with COVID-19 claimed to be unsure about whether they had contact with an infected person or not (Tenforde et al., 2020), raising the possibility that these patients’ lack of information about previous COVID-19 exposures was due to the fact that the exposure happened in environments generally accepted as safe, such as outdoor environments (Clouston et al., 2021). Outdoor social events, such as backyard barbecues, viewing outside events, standing in line, or socializing outdoors, may be sensitive to factors that may affect their protective properties, such as air turnover rate and number of people present (Clouston et al., 2021).

However, most of the studies regarding SARS-CoV-2 RNA presence in air refer to indoor environments and healthcare-related facilities, with only a few studies until this date having reported SARS-CoV-2 RNA presence in outdoor environments (Chirizzi et al., 2021; Dunker et al., 2021; Passos et al., 2021; Pivato et al., 2021; Silva et al., 2023; Tao et al., 2022). Notably, outdoor airborne transmission has been pointed as playing a significant role in the COVID-19 outbreak that occurred in northern Italy in the winter of 2020 (Conticini et al., 2020; Setti et al., 2020a; Setti et al., 2020b).

With that in mind, the aim of this study was to assess the presence of SARS-CoV-2 RNA in outdoor air environments in the city of Valladolid, Castille y León, Spain, during winter 2021, at a time when COVID-19 reported cases were starting to increase in the country (The New York Times, 2023). We also aimed to compare SARS-CoV-2 RNA presence in air at two different settings, crowded outdoor environments versus empty outdoor environments, as this data might be relevant for prevention strategies and public health.

## Materials and Methods

### Sampling Locations

**Fig. 1:**
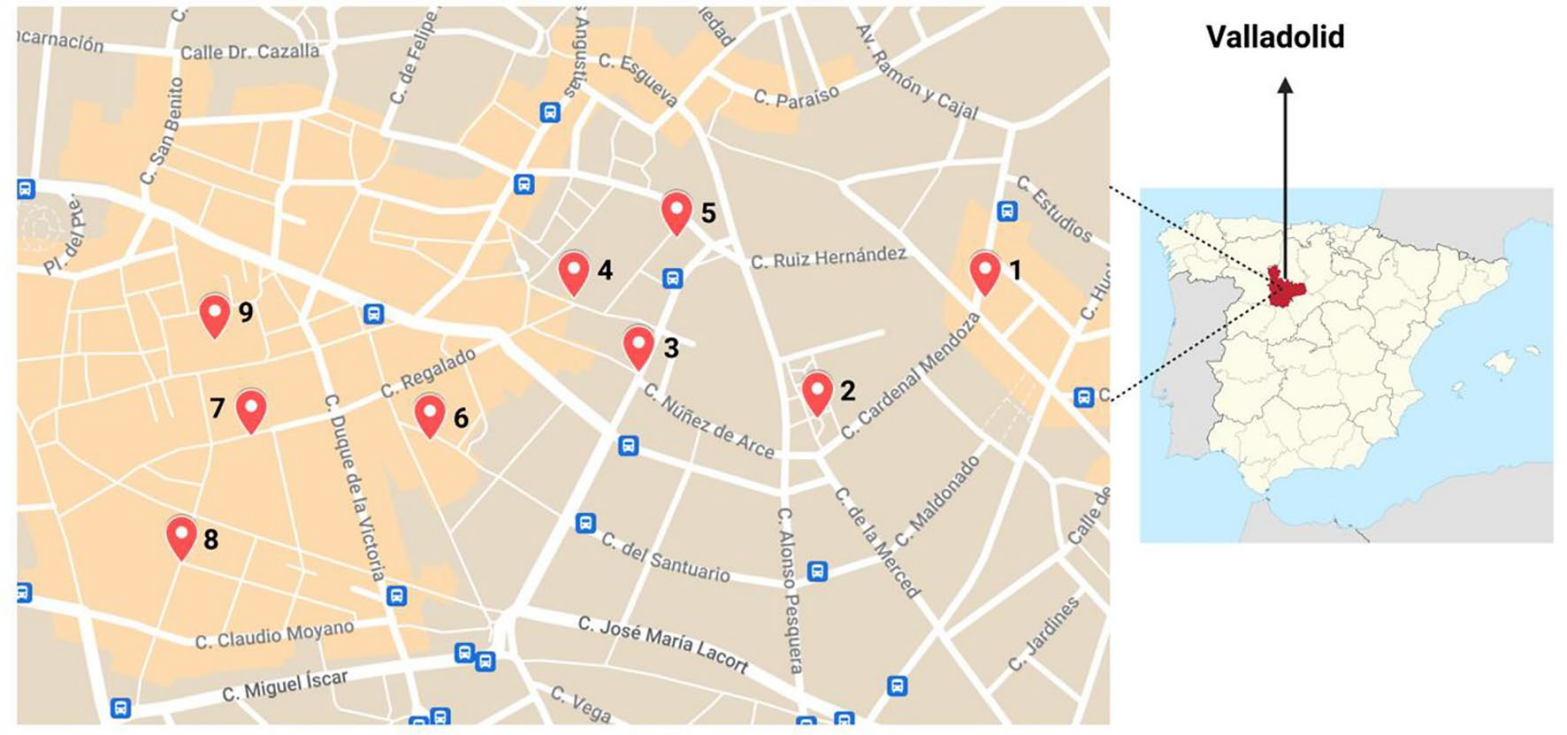
Sampling locations: in the city center of Valladolid: 1—street; 2—public square; 3—street; 4—stairs of a church; 5—university’ square; 6—front of the post-graduate students’ dormitory; 7—commerce street; 8—commerce street; 9—Christmas market at the city’s main square. Figure created with BioRender.com

Air sampling was performed in December 2021, in outdoor areas of the city of Valladolid, Castille y León, Spain. Air samples (*n* = 20) were collected from nine different locations, with each location being sampled twice, namely in the afternoon, between 13:00 and 17:00, when most of the stores were closed and streets were mostly empty, with no people or only one person at most passing by during all the sampling periods—designated from now on as “empty-street scenario” –, and again after this period, between 18:00 and 23:00, when stores were open again and streets had a big influx of people passing by in every direction, with at least 30 people passing by the sampler every minute with less than 1 m distance between them – “crowded-street scenario”. Sampling locations are represented in Fig. 1. At one of the locations (number 9), two samples were collected in each period of the day because it was the place with more density of people. Of note, there were at least 50 people close to the sampler during the whole sampling time on location 9.

### Air Sampling

Air samples were collected using the Coriolis® Compact (Bertin Instruments, Montigny-le-Bretonneux, France) dry cyclonic air sampler with an airflow rate of 50 L/min for 20 min (total of 1 m^3^). The sampler was placed at approximately 1.5 m height in every sampling site. Air samples were collected at first in an empty sterile collection cone. After each sampling, 4 mL of sterile phosphate buffered saline (PBS) (GRISP, Porto, Portugal) was added to the collection cone. All samples were stored at 4^◦^C until transportation to the laboratory facilities and were processed within 24 h.

Following manufacturer directions, the sampler was cleaned and decontaminated after each sampling. Surfactant–water wipes cleaned the Coriolis Compact's exterior. After that, the sampler was wiped clean.

During air sampling KN95 masks and gloves were used by the operator, with masks replaced after each sampled area and gloves after each sample. Hands were properly washed and disinfected between handling of each sample.

### RNA Extraction and RT-qPCR

RNA extractions were performed using the GRS Viral DNA/RNA Purification Kit (GRISP, Porto, Portugal) according to the manufacturer’s instructions. RNA extraction was performed using 200 μL of samples suspensions as previously described (Santarpia et al., 2020). A one-step RT-qPCR targeting the two viral genes N1 and N2 of SARS-CoV-2 was used (Xpert qDetect COVID-19, GRISP, Porto, Portugal). The selected targets, corresponding primer sequences and probes are based on a protocol previously described (CDC, 2020). The CFX Real-Time PCR (qPCR) Detection System (Bio-Rad, USA) with the Bio-Rad CFX Maestro 1.0 Software version 4.0.2325.0418 was used to control the runs and remotely analyze the data. Each RT-qPCR run included ssDNA targets for both N1 and N2 regions (positive controls) and a no-template control. Reactions were set up and run with initial conditions of 15 min at 45 °C and 2 min at 95 °C, then 45 cycles of 95 °C for 15 s and 55 °C for 30 s. A standard curve was generated using ssDNA targets for both N1 and N2 regions, in a tenfold serial dilution mixture starting at 200,000 copies/µL for quantification of the number of viral gene copies present in each samples from the measured Ct values, with a limit of detection (LOD) of 1.3 copies/µL for N1 and 3.2 copies/µL for N2. All samples were tested in duplicates, and air sample results were expressed in copies/uL.

## Results

From the total of the 20 air samples tested, six were positive for SARS-CoV-2 RNA. In the first PCR run, all 14 negative samples did not amplify at all. In the second PCR run for the same samples, 11 did not amplify at all, two amplified with Ct > 38 for one of the targets (which is the cut-off value for the protocol we used), and one amplified only for target N2 with a Ct > 38. Considering the manufacturer’s instructions about the cut-off value, and the fact that they amplified in only one of the PCR runs, we did not consider these three samples as positives.

None of the samples collected during the “empty-street scenario” (period of the day between 13:00 and 17:00) were positive for SARS-CoV-2 RNA, whereas all the positive samples were collected during the “crowded-street scenario” (period of the day between 18:00 and 23:00). Details about the air sampling (date, period of the day, location) and number of SARS-CoV-2 RNA copies of the positive samples are summarized in Table 1.

**Table 1:** Details about location and RT-qPCR results for the positive samples

Sample location number	Date of air sampling	Period of the day	Sampled location GPS coordinates	Ct (N1)	Gene copies/µL (N1)	Gene copies/m^3^ (N1)	Ct (N2)	Gene copies/µL (N2)	Gene copies/m^3^ (N2)
1	03/12/21	13:00–17:00	41.6523930, − 4.7181090	–	–	–	–	–	–
2	03/12/21	13:00–17:00	41.6511800, − 4.7203800	–	–	–	–	–	–
3	03/12/21	13:00–17:00	41.6516460, − 4.7228040	–	–	–	–	–	–
4	03/12/21	13:00–17:00	41.6523970, − 4.7236870	–	–	–	–	–	–
5	03/12/21	13:00–17:00	41.6530230, − 4.7236120	–	–	–	–	–	–
6	03/12/21	13:00–17:00	41.65094, − 4.72564	–	–	–	–	–	–
7	03/12/21	13:00–17:00	41.6509970, − 4.7280660	–	–	–	–	–	–
8	03/12/21	13:00–17:00	41.64971, − 4.729	–	–	–	–	–	–
9	03/12/21	13:00–17:00	41.65195, − 4.72856	–	–	–	–	–	–
10	03/12/21	13:00–17:00	41.65195, − 4.72856	–	–	–	–	–	–
1	03/12/21	18:00–23:00	41.6523930, − 4.7181090	36.65	3.61	45,125	36.75	3.31	41,375
2	03/12/21	18:00–23:00	41.6511800, − 4.7203800	35.77	7.65	95,625	36.77	3.25	40,625
3*	03/12/21	18:00–23:00	41.6516460, − 4.7228040	38.11	–	–	36.82	–	–
4	03/12/21	18:00–23:00	41.6523970, − 4.7236870	37	2.68	33,500	35.93	6.72	84,000
5*	03/12/21	18:00–23:00	41.6530230, − 4.7236120	36.28	–	–	38.90	–	–
6*	03/12/21	18:00–23:00	41.65094, − 4.72564	–	–	–	38.50	–	–
7	03/12/21	18:00–23:00	41.6509970, − 4.7280660	–	–	–	–	–	–
8	03/12/21	18:00–23:00	41.64971, − 4.729	32.38	141.25	1,765,625	32.14	173.34	2,166,750
9	05/12/21	18:00–23:00	41.65195, − 4.72856	35.03	14.52	181,500	36.39	4.51	56,375
10	05/12/21	18:00–23:00	41.65195, − 4.72856	32.53	123.42	1,542,750	32.47	130.41	1,630,125

– negative samples that did not amplify

*excluded samples due to Ct > 38 and/or amplification for only one of the target genes

## Discussion

At the time of this investigation, in early December 2021, there was a rapid increase in COVID-19 cases reports as a result of the rapid spread of the Omicron variant (B.1.1.529) worldwide (Khemiri et al., 2022). This variant had the capacity to infect six times as many people as Delta (B.1.617.2) over the same time and with an Rt of 1.4– to 3.1-fold higher than Delta (Callaway & Ledford, 2021; CDC, 2023; Tian et al., 2022). In addition, the B.1.1.529 Omicron variant showed to be able to propagate more easily among children than the previous variants (Chun et al., 2022).

Studying RNA detection of SARS-CoV-2 in different environments and matrices is crucial from a public health perspective for several reasons. Environmental monitoring, such as detecting SARS-CoV-2 RNA in wastewater and bivalve mollusks, provides an early warning of infection outbreaks in a community before clinical cases are identified, allowing for timely public health interventions to prevent the spread of the virus (Medema et al., 2020). Bivalve mollusks, which can accumulate viruses due to their ability to filter large amounts of water, can serve as indicators of virus presence in aquatic environments (Lombardi et al., 2023). Similarly, understanding the presence of the virus on surfaces and in the air in various environments, like hospitals, public transportation, and schools, helps assess the risk of fomite and airborne transmission, leading to improved infection control measures (Morawska & Cao, 2020; Riddell et al., 2020).

In the current study, the presence of SARS-CoV-2 RNA was assessed in two different epidemiological contexts, “empty-street scenario” *versus* “crowded-street scenario”, namely deserted streets versus busy streets. None of the air samples collected during the “empty-street scenario” were positive for SARS-CoV-2 RNA. However, the findings changed with samples collected during the “crowded-street scenario”, as six samples from this scenario were found positive for SARS-CoV-2 RNA, representing 30% of all the air samples and 60% of samples collected on the “crowded-street scenario” timeframe.

In Spain, it is usual for stores to close between 14:00 pm and 17:00 pm, especially during the summer (Spain’s official tourism website, 2023), with the majority of the streets ending up deserted during these hours. The air sampling campaign happened in December, when Christmas Markets were open and a great number of families with children were present outdoors in these places, contributing to an increased number of people on the streets at night, the timeframe where all the positive samples were collected. Events that attract a large number of people like Christmas markets, typically take place in confined spaces, either indoors or outdoors, for an extended period of time, during which attendees eat and drink in close quarters with one another. These conditions could increase the likelihood of SARS-CoV-2 spread and could destabilize the health system's ability to respond if a large number of individuals become infected (WHO, 2020a).

Environmental variables such as ultraviolet light (UV) exposure, temperature, relative humidity, wind currents, and ventilation systems can also influence air sampling results (Asif et al., 2022; Kumar et al., 2021), with SARS-CoV-2 showing to be considerably more resistant to environmental degradation than other enveloped viruses (Senatore et al., 2021). The environmental conditions in Valladolid during December are characterized by low temperatures ranging from 1.2 °C to 8.5 °C and high humidity levels averaging around 82% (Climate Weather, 2024; Weather Atlas, 2024). During both sampling days, THE average temperature was 9.7 °C and 10.7 °C, respectively, with average relative humidity of 84% and 73%, respectively (TuTiempo.net, 2021). Cold temperatures and high humidity are known to enhance the stability of the virus in aerosols, potentially increasing its persistence and transmissibility in the air (Audi et al., 2020a, 2020b). Moreover, the relatively low UV index (around 2) indicates minimal natural disinfection from UV radiation, which could further contribute to the prolonged viability of the virus on surfaces and in the air (Ratnesar-Shumate et al., 2020).

Moderate wind speeds in Valladolid during December, averaging 10 km/h, might also influence the dispersion of viral particles (Meteoblue, 2024). While higher wind speeds can disperse aerosols over larger areas, potentially reducing concentration, moderate winds may not significantly dilute viral loads in crowded outdoor settings (Dabisch et al., 2021). During the two sampling days, average wind speed was 21.4 and 20.8 km/h, respectively (TuTiempo.net, 2021). The combined effect of these factors suggests that winter conditions in Valladolid might facilitate airborne transmission of SARS-CoV-2, emphasizing the importance of indoor ventilation and adherence to public health measures during this season.

In a systematic review about the transmission of SARS-CoV-2 and other respiratory viruses in outdoor environments, authors concluded that the existing data support that outdoor environments likely pose a much smaller risk of SARS-CoV-2 transmission than indoor environments, mainly because outdoor environments allow for more physical distancing and better airflow and ventilation and less recycled air, all of which help to minimize airborne transmission (Bulfone et al., 2021b). In another study in which a simplified analytical model to compare the relative level of exposure occurring between comparable outdoor and indoor settings was developed the authors concluded that the risk of transmission outdoors is considerably lower than indoors (Rowe et al., 2021). However, authors adverted to situations of temperature inversion (when cool air is trapped at the ground beneath a layer of warm air) and low wind speeds, as it could result in levels of outdoor transmission close to those indoors, especially in crowded spaces (Rowe et al., 2021). In a study carried out in the municipality of Borriana, Spain, that attempted to calculate the odds of an outbreak of COVID-19 that occurred there and its correlation with large public gatherings, it was found that exposure during events where food and dancing took place played a major factor in the spread of COVID-19 (Domènech-Montoliu et al., 2021).

When looking at the findings of the present study, they are consistent with reports stating that risk of infection is lower outdoors, with SARS-CoV-2 RNA presence in air being directly related to the number of people present. In fact, when sampling took place with zero or only one person around, no SARS-CoV-2 RNA was detected, and when sampling took place in heavily crowded streets, where physical distancing of at least 1 m or more was not possible, more than half of the samples were positive for SARS-CoV-2 RNA.

Although the minimum infectious dose for SARS-CoV-2 in humans still hasn’t been determined, it is generally assumed to be low due to the high transmissibility of the virus (Karimzadeh et al., 2021). Research in hamsters suggests that low viral loads such as 10^3^ or 10^5^ 50% tissue culture infective doses (TCID_50_) could be sufficient to initiate infection with SARS-CoV-2 (Imai et al., 2020). Considering that the number of RNA copies/m^3^ found in this study ranged from 2680 to 173,590, and presuming that the virus was still viable, would indicate a non-negligible risk of transmission, particularly in settings with high human density and limited ventilation. This is especially relevant considering that SARS-CoV-2 can remain viable and infectious in aerosols for extended periods under certain environmental conditions, as well as the fact that environments with lower temperatures and high humidity might be conducive to virus stability and transmission (Karimzadeh et al., 2021).

In addition to that, it is also important to highlight that in all locations where SARS-CoV-2 RNA was detected in air, there was a great number of children present, which constitutes a vulnerable group that requires attention, especially considering the emergence of more transmissible variants such as Omicron (Khemiri et al., 2022). SARS-CoV-2 RNA detection in air samples from environments with more children present was not unexpected, as increased vaccination rates in the general population usually results in an increase in infection rates among the unvaccinated parcel of the population such as children (Rotevatn et al., 2023).

The risk of acquiring SARS-CoV-2 infections in outdoor environments should not be overlooked if we consider that there is a lack of knowledge regarding the importance of outdoor environments as potential pathways for SARS-CoV-2 infection (Bulfone et al., 2021a). Elucidating more details of the dynamics of SARS-CoV-2 transmission outdoors is of particular interest in high-density cities, where, according to a first-principles model, the transmission of SARS-CoV-2 outdoors is suspected to have played a role in superspreading events (Huang et al., 2021)—whether these environments are crowded and if social distancing cannot be maintained, as well as the increased probability of being in contact with contaminated surfaces or items (fomites) and the number of asymptomatic people present (Senatore et al., 2021) are some examples of important details in order to elucidate the real dynamics of the virus in outdoor air.

Collectively, these results emphasize the importance of preventive measures such as wearing masks in order to avoid SARS-CoV-2 infection, even in outdoor settings. In addition to that, wearing masks can be an important tool to prevent the spread of other respiratory viruses such as respiratory syncytial virus (RSV) and influenza virus (flu), especially during winter when these viruses tend to circulate at the same time (Audi et al., 2020c; Furlow, 2023; Plantinga et al., 2023), resulting in a significant increase in respiratory disease burden worldwide in both high- and low-income countries (Ndumwa et al., 2022; Robinson, 2008; Salvi et al., 2018).

This study has some limitations that should be considered. Firstly, the small sample size may limit the generalizability of the findings, as only a limited number of samples were taken from two specific outdoor settings. Additionally, the study was conducted during the winter season in Valladolid, Spain, when environmental conditions like temperature, humidity, and UV exposure could affect viral persistence and detection differently compared to other seasons. These factors make it challenging to extend the results to other times of the year or geographical locations with varying climatic conditions. Further research incorporating a broader range of environmental conditions and viral viability assessment would enhance our understanding of SARS-CoV-2 behavior in outdoor air.

## Conclusion

While outdoor environments generally pose a lower risk for SARS-CoV-2 transmission than indoor settings, this study detected SARS-CoV-2 RNA in air samples collected from crowded outdoor areas, suggesting that virus presence in the air is influenced by the number of people present. However, the study has limitations, including the small sample size and the fact that it was carried out in winter conditions in Europe, which cannot be generalized to other seasons or locations. Future studies about SARS-CoV-2 in outdoor are that include assessment of viral viability as well as environmental conditions such as humidity, temperature, UV radiation and wind speed are still needed, as these data would help us understand how different environmental factors at play can affect infectivity over time in different types of outdoor environments. In light of the COVID-19 pandemic, and in view of future worldwide respiratory viral pandemics that might surge, it is important for policy-makers and government authorities to promote education for the population about the importance of wearing good quality masks, as well as to provide a wider availability of these types of masks for the population in an accessible manner, as this measure alone could greatly reduce risk of acquiring respiratory infections not only in indoors but also in outdoors environments.

## Data Availability

The data presented in this study are available upon request to the corresponding author.
